# A study on the bio-applicability of aqueous-dispersed van der Waals 1-D material Nb_2_Se_9_ using poloxamer

**DOI:** 10.1038/s41598-020-80730-2

**Published:** 2021-01-08

**Authors:** Sudong Chae, Seungbae Oh, Kyung Hwan Choi, Jin Woong Lee, Jiho Jeon, Zhixiang Liu, Cong Wang, Changmo Lim, Xue Dong, Chaeheon Woo, Ghulam Asghar, Liyi Shi, Joohoon Kang, Sung Jae Kim, Si Young Song, Jung Heon Lee, Hak Ki Yu, Jae-Young Choi

**Affiliations:** 1 School of Advanced Materials Science and Engineering, Sungkyunkwan University, Suwon, 16419 Republic of Korea; 2 SKKU Advanced Institute of Nanotechnology (SAINT), Sungkyunkwan University, Suwon, 16419 Republic of Korea; 3 Research Center of Nanoscience and Nanotechnology, Shanghai University, Shanghai, 200444 China; 4 Biomedical Institute for Convergence at SKKU (BICS), Sungkyunkwan University (SKKU), Suwon, Suwon, 16419 Republic of Korea; 5 Department of Orthopaedic Surgery, Dongtan Sacred Heart Hospital, Hwaseong, Republic of Korea; 6 Department of Materials Science and Engineering, Department of Energy Systems Research, Ajou University, Suwon, 16499 Republic of Korea

**Keywords:** Synthesis and processing, Tissues

## Abstract

In this research, dispersion of a new type of one-dimensional inorganic material Nb_2_Se_9_, composed of van der Waals bonds, in aqueous solution for bio-application study were studied. To disperse Nb_2_Se_9_, which exhibits hydrophobic properties in water, experiments were carried out using a block copolymer (poloxamer) as a dispersant. It was confirmed that PPO, the hydrophobic portion of Poloxamer, was adsorbed onto the surface of Nb_2_Se_9_, and PEO, the hydrophilic portion, induced steric hinderance to disperse Nb_2_Se_9_ to a size of 10 nm or less. To confirm the adaptability of muscle cells C2C12 to the dispersed Nb_2_Se_9_ using poloxamer 188 as dispersant, a MTT assay and a live/dead assay were performed, demonstrating improvement in the viability and proliferation of C2C12 cells.

The development of nanotechnology has led to the study of a variety of inorganic nanomaterials and has been used extensively for the study of low-dimensional materials (e.g., nano-thick two-dimensional (2D) materials such as graphene and one-dimensional (1D) linear materials such as nanowires)^1^. Based on this, nanomaterials have begun to be used in various applications such as electronic devices^2^, optical devices^3^, catalysts^4^, environment technology^5^, and biotechnology^6^. In particular, bio-applications have been utilized in various fields in in-vitro (e.g., electrical and optical bio sensors^7,8^) as well as in-vivo (e.g., plasmonic photothermal therapy^9^, magnetic resonance imaging^10^) environments based on the characteristics of various inorganic materials, such as superior durability, and electromagnetic and optical properties that cannot be found in the organic materials constituting the existing living body.

However, as the size of existing nanomaterials decreases, the ratio of surface dangling bonds to the nanomaterial size increases, and the characteristics of the material properties expected in the bulk state are degraded (e.g., decrease in electron mobility, optical damping, change in magnetic susceptibility)^11^. To solve these problems, 2D nanomaterials based on van der Waals (vdW) bonding without surface dangling bonds have been studied. However, the 2D material is a plate-like structure, which still contains dangling bonds at the edges, and there are structural limitations to having various sizes and shapes for bio-application^12^. Therefore, to apply inorganic nanomaterials to the biotechnology field, novel materials are needed; accordingly, 1D vdW materials have started to receive much attention in recent years.

There are two types of 1D vdW structures; a true 1D structure (e.g., hexagonal tellurium^13^, Mo_6_S_*x*_I_9-*x*_^14^, VS_4_^15^, Nb_2_Se_9_^16^, Ta_2_Pd_3_X_8_^17^ (X = S, Se), Sb_2_Se_3_^18,19^, and V_2_Se_9_^20^) with a pure vdW inter-chain bond and a quasi-1D structure (e.g., transition metal trichalcogenides (TiS_3_^21^, TaSe_3_^22^, ZrSe_3_^23^, NbSe_3_^24^, etc.), Nb_2_PdS_5_^25^, and CsBi_4_Te_6_^26^) with additional non-vdW bonds among molecular chains. In the case of the quasi-1D structure, it is difficult to separate molecular chains that contain bonds other than the vdW variety and control their size and shape. However, in the case of the true 1D structure, it is possible to fabricate an atomic-scale inorganic nanowire without dangling bonds through mechanical separation^27–29^ or chemical dispersion^29–31^. In our research group, we have synthesized a large number of true 1D materials and published the results of separating them into atomic units^20,31^. These materials have excellent surface stability because they have no surface dangling bonds, and their nanostructures of various shapes and sizes are easy to fabricate. Thus, they are likely to be applied to biotechnologies, such as extracellular matrix fabrication^29^, tissue engineering^32^, and drug delivery^33^. To apply this true 1D material to the biotechnology field, first, aqueous dispersion must be performed, and the dispersed material should be biocompatible with no toxicity^34^. However, it is known that the 1D material surface composed of vdW bonds has a very small dipole moment, owing to its nature, and a hydrophobic surface; thus, aqueous dispersion is very difficult. Therefore, it is very important to design a dispersant suitable for aqueous dispersion.

In this study, we used poloxamers as a dispersing agent to disperse the new types of true-1D Nb_2_Se_9_ (see Fig. 1a for crystal structure), which can be mass-synthesized at the cm-scale by a flux method. Nb_2_Se_9_ is composed of strong intra-chain bonds and weak inter-chain interactions of molecular unit chains. Poloxamers are the ABA (PEO-PPO-PEO) type block-copolymer. ABA triblock copolymer has a typical dispersant structure as a polymer having a portion B adsorbed on the surface of a nanomaterial and a portion A that provides affinity with a solvent. They exhibit good hydrophilicity through PEO at both ends and can be dissolved in water. PPO in the middle can be hydrophobic and adsorbed onto the hydrophobic surface of Nb_2_Se_9_. That is, the dispersants can be adsorbed through the hydrophobic-phobic interaction between PPO and Nb_2_Se_9_, and the dispersion can proceed through the steric hinderance of the PEO tail (see the schematic in Fig. 1b). Furthermore, ether groups such as PEO and PPO have excellent biocompatibility. First the effect of Poloxamer structure was investigated for dispersing Nb_2_Se_9_ in deionized water, and PBS buffer solution, similar environment with real biological environment. The morphology and chemical state of exfoliated Nb_2_Se_9_ was characterized by AFM, TEM and XPS. Furthermore, biocompatibility of Nb_2_Se_9_ dispersed with poloxamer 188 was verified using the MTT assay and the proliferation test of C2C12 cells. Biocompatible and water dispersible Nb_2_Se_9_ has the great potential as a building block to make biomaterials for drug delivery, bioimaging, and theragnostic.

**Figure 1:**
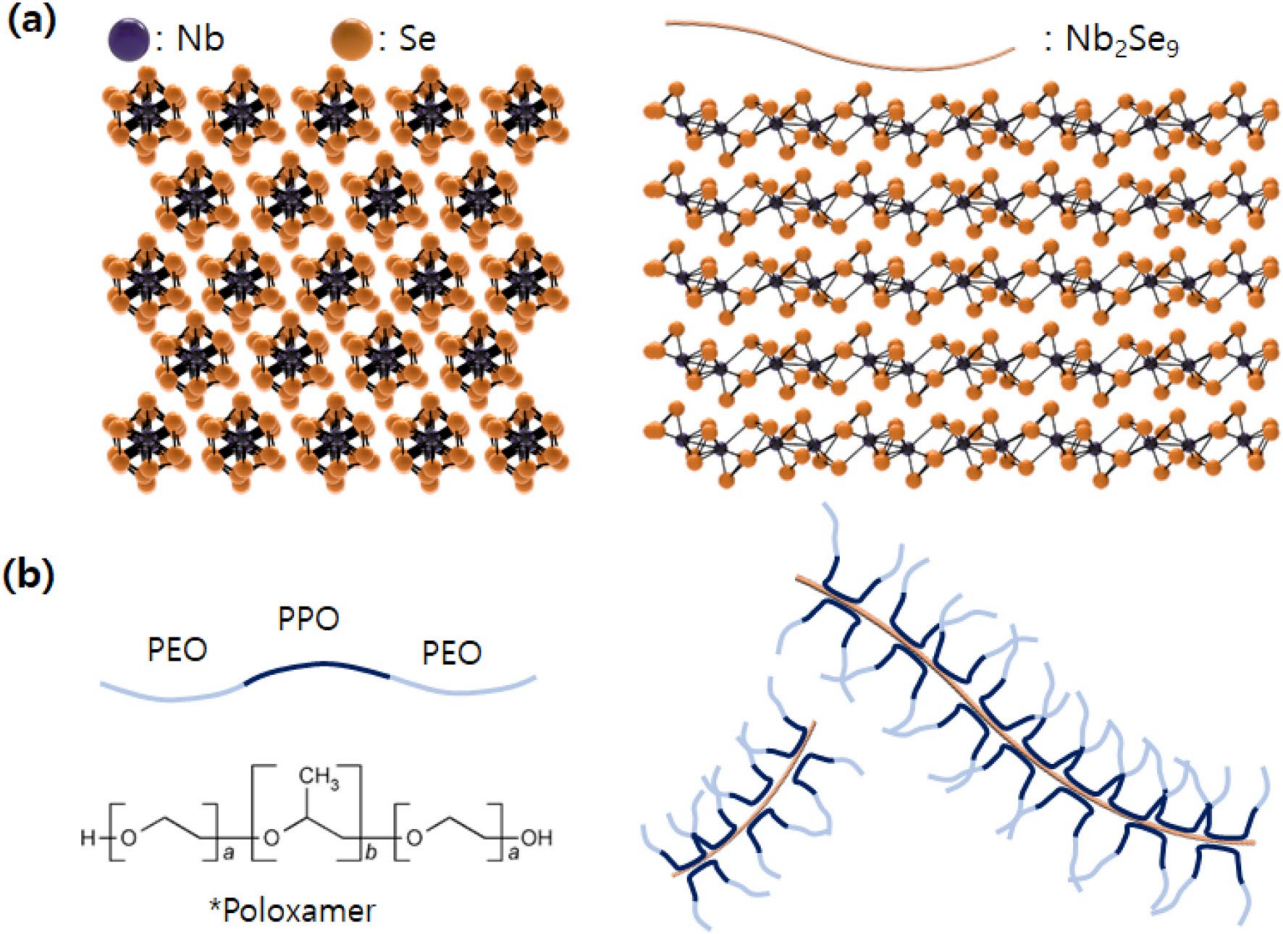
Schematics of (**a**) Nb_2_Se_9_ and (**b**) dispersion of Nb_2_Se_9_ by poloxamer dispersant (PPO part of poloxamer attached to Nb_2_Se_9_ surface and dispersion obtained by the steric hinderance of PEO chains).

## Result/discussion

**Figure 2:**
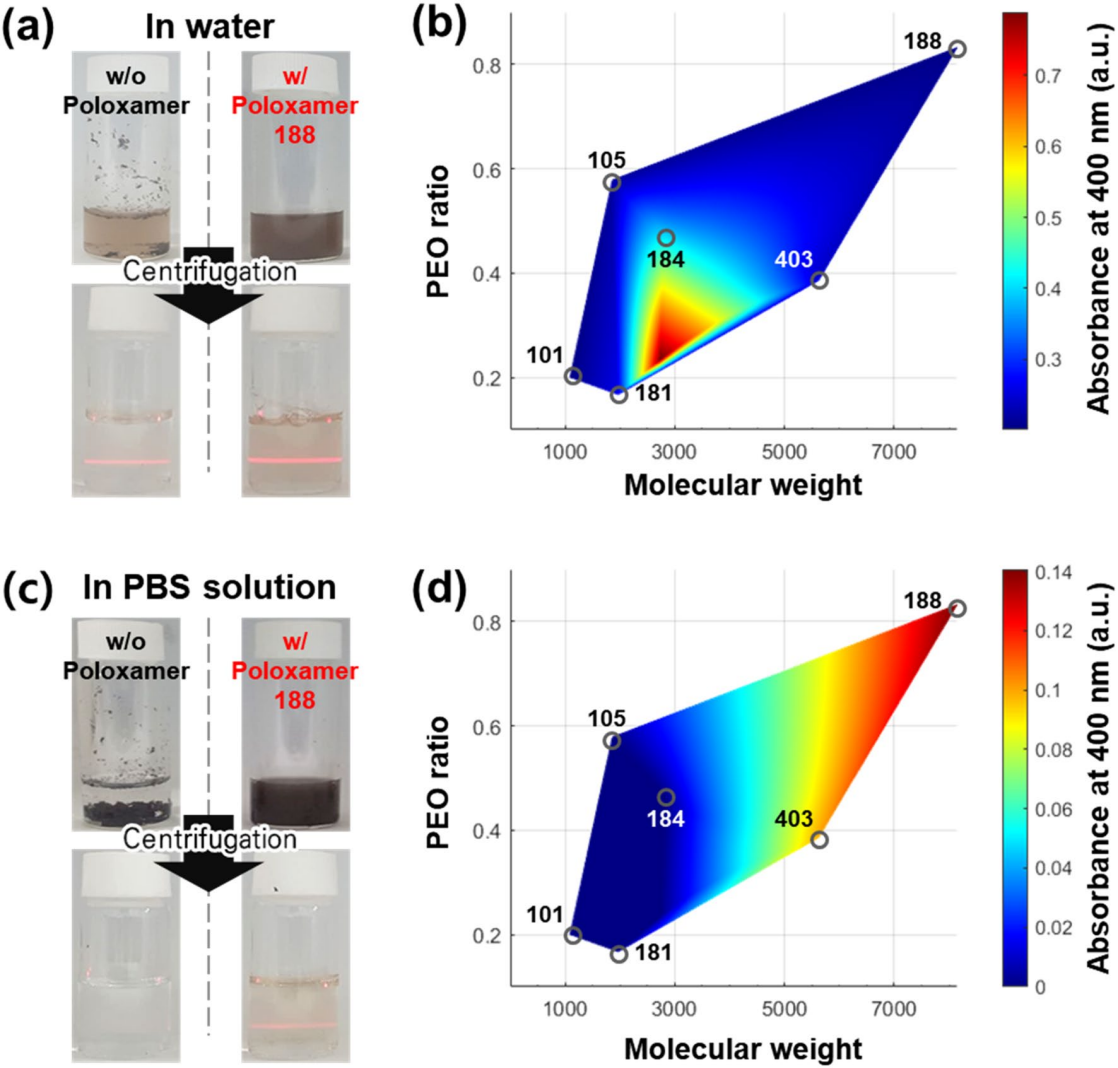
Digital photographs of dispersion solutions (**a**, top: before ultracentrifugation, bottom: after ultracentrifugation) and concentration map of Nb_2_Se_9_ in deionized water dispersed by poloxamer as a function of the molecular weight and the ratio of PEO (**b**). Digital photographs of dispersion solutions (**c**, top: before ultracentrifugation, bottom: after ultracentrifugation) and concentration map of Nb_2_Se_9_ in PBS buffer solution dispersed by poloxamer as a function of the molecular weight and the ratio of PEO (**d**).

In order to study the effect of poloxamer structure, six poloxamers having different numbers of PO and EO chains was adapted (see the Fig. S1). Optical measurements (UV–Vis absorption and Tyndall effect) were performed to verify the aqueous dispersion performance of Nb_2_Se_9_ using poloxamers. First, starting material, Nb_2_Se_9_ crystal is immersed in the water with poloxamer and ultrasonication is carried out on it. Centrifugation removes the undispersed portion and the laser irradiation reveals a pronounced Tyndall effect and increased UV–Vis absorption. When Poloxamer was not used, the Nb_2_Se_9_ was not dispersed and sunk to the bottom of the water. However, when the poloxamer dispersant is used, uniform dispersion proceeds without residue (see the strong Tyndall effect in the Fig. 2a and Fig. S2). In deionized water, the dispersion of Nb_2_Se_9_ nanowire dispersed by various poloxamers was stable after 1 week.

To compare the dispersibility using various poloxamers, the intensity of the characteristic UV–Vis absorption peak near 400 nm in the solutions after centrifugation was plotted (see the Fig. 2b,d) Well-dispersed Nb_2_Se_9_ nanowires have a broad UV–Vis absorption at 350–650 nm and shoulder peaks at 400 and 570 nm^35^(see the Fig. S3 and S5). As shown in Fig. 2b, the aqueous dispersion of Nb_2_Se_9_ was stable using the poloxamers with molecular weight of approximately 3000. When the molecular weight is too small, the steric hinderance was not enough to stabilize the Nb_2_Se_9_ wires in water due to its short PEO chain. In addition, when the molecular weight is too large, the poloxamer cannot be adsorbed effectively on the surface of Nb_2_Se_9_ chains.

In PBS solution, the improvement in dispersion of Nb_2_Se_9_ through poloxamer is more pronounced than in pure water. In the PBS solution without the poloxamer, Nb_2_Se_9_ was hardly dispersed and mostly sunken, while the dispersibility was clearly improved with the poloxamer (Fig. 2c and Fig. S4). In PBS buffer solution, the dispersion stability of Nb_2_Se_9_ is improved as the molecular weight and the ratio of PEO chain increased (Fig. 2d). Nb_2_Se_9_ nanowires dispersed with poloxamer 101, 105, 181, and 184 whose molecular weight is less than 3,000 were hardly dispersed, showing very weak Tyndall effect and low UV–Vis absorption at 400 nm. On the other hand, the Nb_2_Se_9_ dispersed solution dispersed with poloxamers 188 and 403 having a molecular weight of more than 5,000 showed strong Tyndall effect and UV–Vis absorption at 400 nm. The solution dispersed with poloxamer 188 with a longer hydrophilic PEO tail was able to disperse Nb_2_Se_9_ at a higher concentration than the solution with poloxamer 403, and both dispersions with poloxamer 188 and 403 were stable for 1 week. This is due to the compression of electrical double layer by present ions in the buffer solution. As the electrical double layer is compressed, stronger steric hinderance is needed to stabilize the dispersion of Nb_2_Se_9_ in the PBS solution. Therefore, the poloxamers which have more PEO chains and large molecular weight such as poloxamer 188 and 403 were effective. In the zeta potential analysis of the dispersed solution, short poloxamers such as 101, 105, 181, and 184 had a zeta potential of − 30 to − 35 mV because they could hardly screen the surface charge (− 37 mV) of Nb_2_Se_9_ (see the Fig. S6). On the other hand, the long poloxamers such as 188 and 403 screened the surface charge of Nb_2_Se_9_ and had a zeta potential of − 20 mV similar to the surface charge of ethylene oxide. Even though the electrostatic charge was smaller in the dispersion solution using the long poloxamer, the result of showing more excellent dispersibility is that the effect of steric repulsion was greater than that of electrostatic repulsion on the dispersion of Nb_2_Se_9_ in the electrolyte. Improving the dispersibility of Nb_2_Se_9_ through the poloxamer 188 on PBS solution facilitates its more effective utilization in the real-life environment. Accordingly, the Nb_2_Se_9_ dispersed with poloxamer 188, which showed the best dispersibility in the electrolyte, was applied for further analysis.

**Figure 3:**
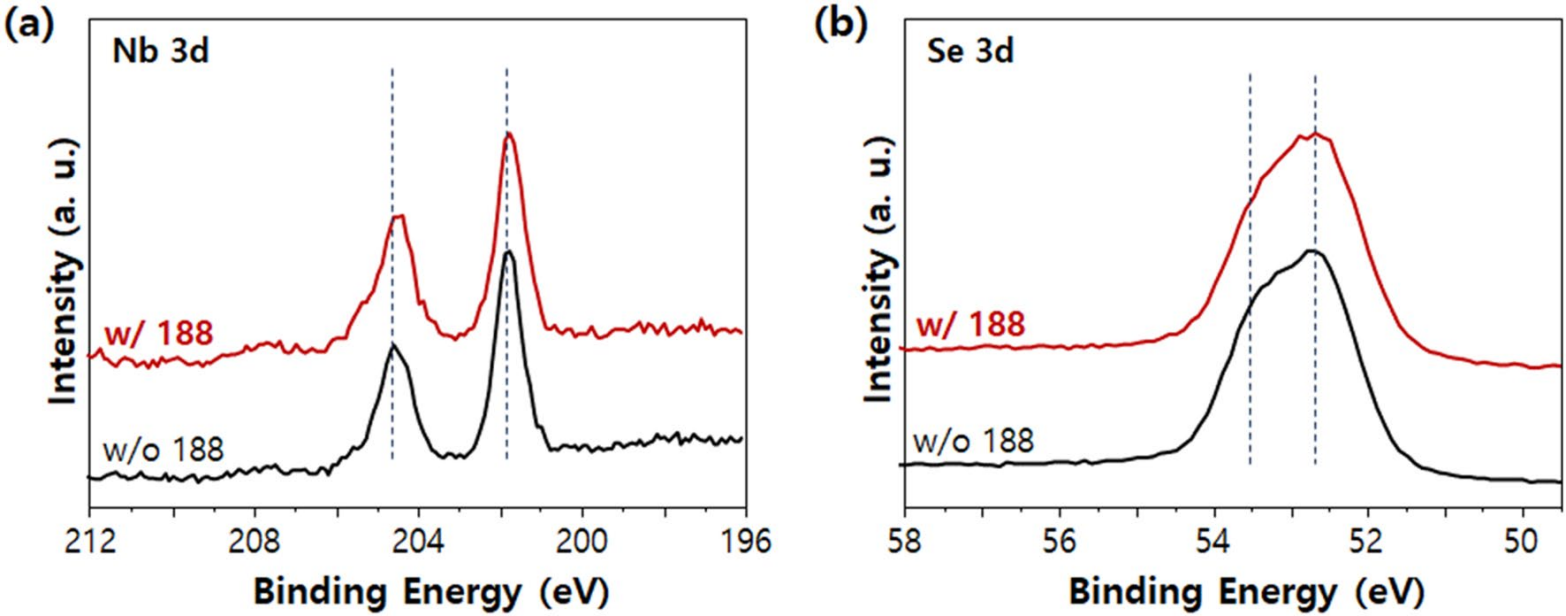
XPS core level spectra of Nb_2_Se_9_ (**a**) Nb 3d and (**b**) Se 3d with respect to use of poloxamer 188.

The enhanced dispersibility of Nb_2_Se_9_ through the poloxamer 188 is due to the hydrophobic-phobic interaction between the PPO chains and Nb_2_Se_9_ surfaces, and the steric hinderance of PEO chains. That is, to confirm the phenomenon of PPO physisorption on the surface of Nb_2_Se_9_, the XPS analysis of Nb_2_Se_9_ surface dispersed by the poloxamer 188 was performed. As can be seen from the core level spectra of Nb *3d* and Se *3d* (Fig. 3a,b), there was no change in the peak shape or binding energy according to the use of poloxamer 188 dispersant (If the electron is moved from the poloxamer 188 to Nb_2_Se_9_, the lower binding energy shift, or from Nb_2_Se_9_ to the poloxamer 188, higher binding energy shift)^35^. This indicates that electron transfer and new compound formation reaction did not occur between the PPO of poloxamer 188 and the Nb_2_Se_9_ surface; rather, only physisorption occurs.

**Figure 4:**
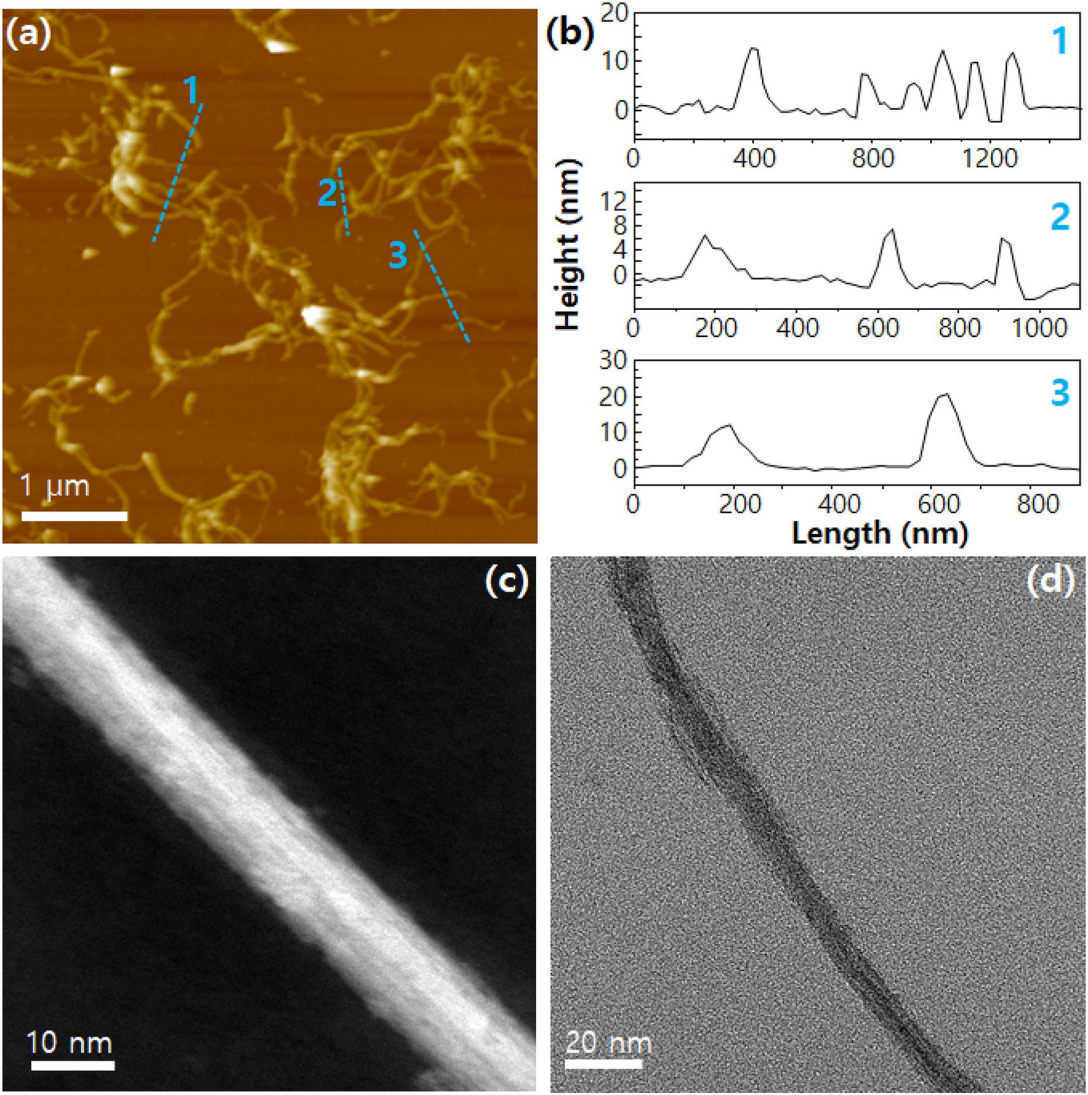
(**a**) AFM images of the dispersed Nb_2_Se_9_ by poloxamer 188. (**b**) Height profiles of dashed lines in Fig. 4a. (**c**, **d**) TEM image of dispersed Nb_2_Se_9_ by poloxamer 188.

The size of Nb_2_Se_9_ dispersed by poloxamer 188 was analyzed by AFM (See the Fig. 4a and 4b). It can be confirmed that the 1D-structured Nb_2_Se_9_ chains with an average diameter of ~ 10 nm were well dispersed (minimum diameter was ~ 6 nm). Owing to large molecular weight of poloxamer 188 molecules, the dispersant is difficult to remove completely, resulting in residue in the AFM images (this phenomenon is common in other previous studies using polymer dispersants)^36^. The 10-nm-thick dispersed Nb_2_Se_9_ nanowires are shown in the TEM images in Fig. 4c and 4d (residue of polymer dispersant can also be seen).

**Figure 5:**
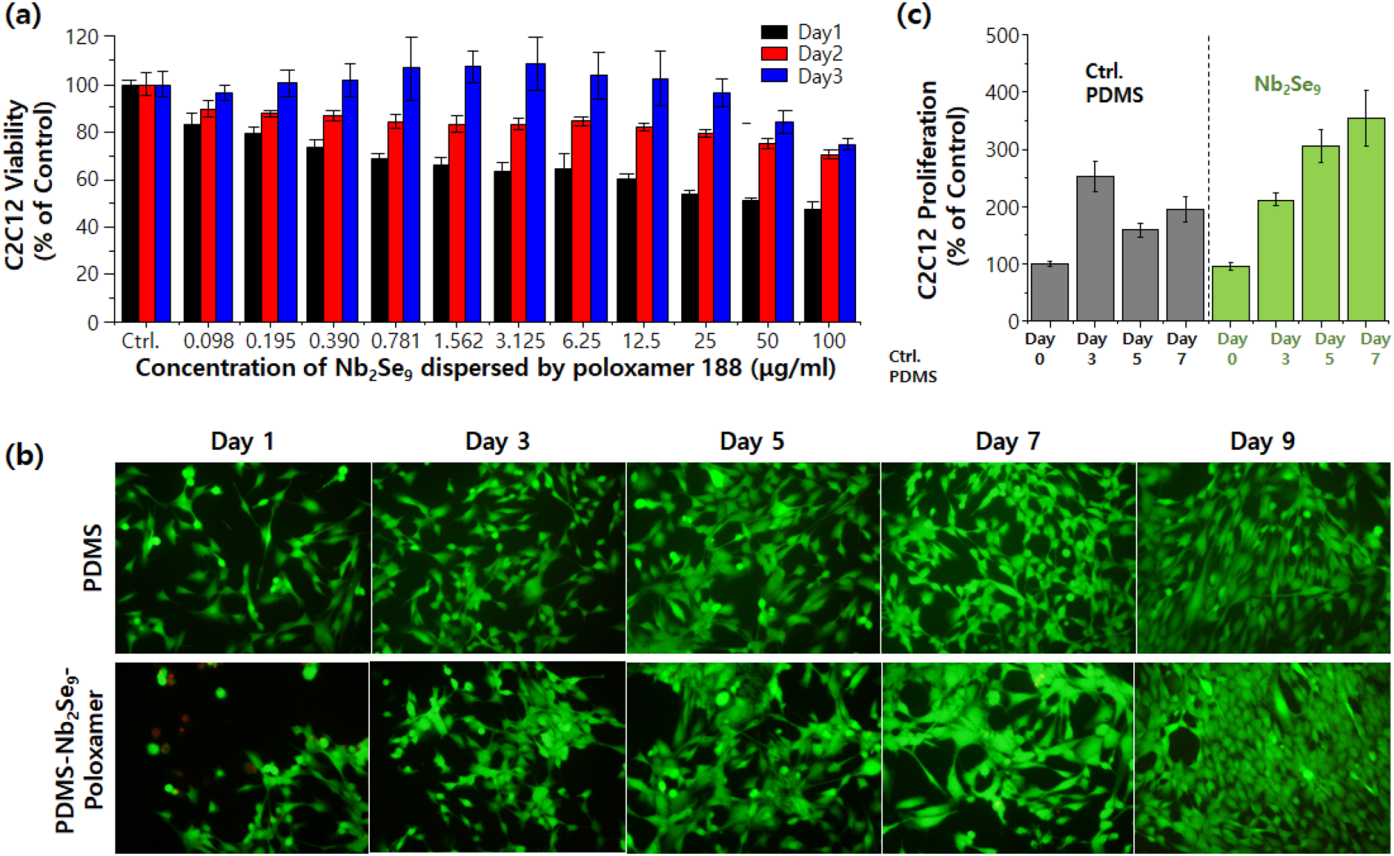
Cytotoxicity of Nb_2_Se_9_ on C2C12 cells (**a**) by MTT assay test with respect to dispersion concentration Nb_2_Se_9_ in water over three days, (**b**) by Live/Dead assay on PDMS substrate over nine days. (**c**) Long-term biocompatibility of Nb_2_Se_9_ on C2C12 by MTT assay using PDMS substrate over seven days (compared with pure PDMS as control device).

For the muscle tissue cells such as C2C12, the nanofibrous structure of extracellular matrix (ECM), which the Nb_2_Se_9_ nanostructures dispersed by the poloxamer 188 would mimic, is the most important issue. To evaluate the cytotoxicity of Nb_2_Se_9_, the MTT assay was performed (Fig. 5a). Although low viability is demonstrated as the treated concentration of Nb_2_Se_9_ on C2C12 is increased, it was recovered very quickly, within 24 h, owing to the interaction of C2C12 with the Nb_2_Se_9_ nanofibrous structure, which resembles its own ECM. For the long-term cytotoxicity, live/dead assay was also performed (Fig. 5b). The nanofibrous Nb_2_Se_9_-coated PDMS was compared with bare PDMS (FDA approved material with sufficient biocompatibility to be implanted in human body) control in this experiment. After 9 d, the proliferation of C2C12 on Nb_2_Se_9_-coated PDMS is enhanced compared with bare PDMS, indicating a greater filling area in the live/dead assay. Thus, the nontoxicity of the Nb_2_Se_9_ in the C2C12 cell line is confirmed by both the MTT and Live/Dead assay. To check the long-term biocompatibility of Nb_2_Se_9_ on C2C12, the MTT assay was selected (Fig. 5c). MTT assay performed on PDMS and the Nb_2_Se_9_-coated PDMS in the same way as the Live/Dead assay. The cell proliferation of C2C12 was normalized by day 1 for each PDMS film (bare and Nb_2_Se_9_-coated PDMS). The Nb_2_Se_9_-coated PDMS shows a growth of 354.5 ± 47.91% in 168 h of culturing (linear increase with time), compared with 195.3 ± 22.63% for bare PDMS (even lower than in 72 h of culturing). These results explain that the nanofibrous Nb_2_Se_9_-coated film provides good conditions for C2C12 cell growth.

## Conclusion

In conclusion, Nb_2_Se_9_, a new 1D material based on vdW binding, was dispersed in water and applied to the ECM of muscle cells C2C12 for biotechnology applications. To disperse hydrophobic Nb_2_Se_9_ in water and PBS solution, poloxamers (a block copolymer in which PEO-PPO-PEO units are polymerized in an ABA structure) were used as a dispersant. It was confirmed that PPO, the hydrophobic portion of poloxamer, was adsorbed onto the surface of Nb_2_Se_9_, and PEO, the hydrophilic portion, induced steric hinderance to disperse Nb_2_Se_9_ to a size of 10 nm or less. Both an MTT assay and a live/dead cell assay were performed to evaluate the viability and proliferation of C2C12 in Nb_2_Se_9_ dispersed using poloxamer 188. The Nb_2_Se_9_-coated PDMS shows a growth of 354.5 ± 47.91% in 168 h of culturing, compared with 195.3 ± 22.63% for control-PDMS. It has been demonstrated that these vdW 1D materials can be used for various bio-related applications if the dispersion in water is efficient.

## Methods

### Preparation of Nb_2_Se_9_ crystal

The preparation of Nb_2_Se_9_ was prepared as previous report^37^. Single-crystalline Nb_2_Se_9_ was synthesized using the flux method in an evacuated quartz tube. The pelletized 0.2 g of Nb (325 mesh, 99.5%, Sigma-Aldrich) and 34 g of Se (99 + %, Alfa Aesar) mixture in a molar ratio of Nb:Se = 1:200 was sealed in an evacuated quartz tube in a 15-cm-long quartz tube with a neck in the middle of the tube. The quartz ampoule was heated for 3 days at a temperature of 700 °C and then cooled. After cooling naturally, the unreacted Se was roughly separated by turning the quartz tube over and dropping the Se liquid to the bottom side of the tube at 250 °C. The resulting material has a dark gray needle shape. The unreacted Se was completely sublimated by heat treatment in a tube furnace at 250 °C under Ar atmosphere for 24 h.

### Preparation of aqueous dispersed Nb_2_Se_9_

To obtain aqueous dispersion, various types of triblock copolymer with different length of PEO and PPO chain, poloxamers (Sigma-Aldrich) were used as a dispersant. First, 10 mg of dispersant, poloxamer, was dissolved in 10 mL of deionized water or 1X PBS solution. After immersing 10 mg of single-crystalline Nb_2_Se_9_ in the solution, ultrasonication was performed by the probe sonication (VC 505, Sonics & Materials, Inc.) at intervals of 2 s on / 2 s off for 5 min, followed by 3 h of bath sonication (B2005S-68 K, 68 kHz, 200 W, KODO Technical)^35^. After sonication, the solution was centrifuged at 6000 rpm for 10 min to remove any insufficiently exfoliated chains, and 5 ml of the supernatant was used for further analysis.

### Characterization

To compare the concentration of dispersed solutions, their UV absorption were characterized by UV–Vis spectrophotometer (Agilent Technologies lnc., Agilent 89090A). The zeta potential of dispersed solution was investigated by Zetasizer (Malvern Instruments Ltd., Nano-ZS90). The chemical state of the Nb_2_Se_9_ was examined by XPS (Thermo, ESCALAB250). The samples for XPS were prepared by vacuum filtration method on the AAO membrane with a pore diameter of 100 nm. The morphologies of the exfoliated nanowires were analyzed by atomic force microscopy (Park Systems, NX 10) and aberration-corrected scanning transmission electron microscopy (STEM, JEOL, JEM-2011F). The AFM was operated in non-contact mode, and the samples for AFM were prepared on SiO_2_/Si wafers by spin-coating. The samples for STEM were prepared by drop-casting onto a carbon-coated TEM grid^35^.

### Cell lines

The C2C12 cell line was obtained from the Korean Cell Line Bank (KCLB, Seoul, Republic of Korea). The cells were cultured in Dulbecco’s modified Eagle’s medium (DMEM, PAN biotech), supplemented with 10% fetal bovine serum (JCBIO) and 1% penicillin–streptomycin (PAN biotech). The cells were incubated at 37 °C in a standard atmosphere (95% air and 5% CO2) during all experiments. The cell culture was maintained with 9 × 105 cells in 75-cm2 cell culture flasks (SPL Life Sciences), and sub-cultured every 48 h after seeding by replacing the culturing media every day. The cell was cultured for three passages after thawing to stabilize the growth conditions^29^.

### Proliferation of C2C12 with Nb_2_Se_9_ dispersed by poloxamer 188

The 5 × 10^3^ cells were seeded 24 h before the experiment for normalization in each well of a 96-well plate (SPL Life Sciences). The solution of Nb_2_Se_9_ dispersed by poloxamer 188 (Nb_2_Se_9_-188) was prepared in 1X PBS (Gibco), and subsequently, a UV-sterilized procedure was conducted before mixing with the complete media. The dispersed solution was condensed to the required concentration using an Amicon filter. The Nb_2_Se_9_-188 was mixed with complete media (1:9 vol%) to obtain the final concentration, and 200 μL of the mixed media was treated to C2C12 in each 96-well plate. After the required incubation time passed, 0.5 mg/mL of filtered thiazolyl blue tetrazolium bromide (MTT) in serum-free media was replaced with previous culturing media in a 96-well plate. C2C12 with the MTT solution was incubated for 3 h in 37 °C and replaced with dimethyl sulfoxide (DMSO) for the lysis and dissolution of formed formazan. A spectrophotometer (Varioskan LUX) was used to obtain the absorbance of 570 nm and 630 nm of the samples. The proliferation was calculated by subtracting the absorbance of 630 nm from 570 nm in each sample^29^.

### Substrate fabrication

The substrate and wells of the PDMS well plate were fabricated using SYLGARD 184 Base & Curing Agent Kit (Dow Corning). The mold to fabricate the PDMS well plate was made by PTFE and glass. The PTFE and glass mold were washed by sonication in ethanol, methanol, and DI water for 10 min each. Particularly for the glass plate, trichlorosilane (Tokyo Chemical Industry) was coated to give hydrophobicity on the glass plate, enhancing the PDMS molding technique. The PDMS well plates were made into a 24-well plate size. The PDMS prepolymer and curing agent were mixed in a 10:1 ratio and degassed in a vacuum oven. The PDMS substrates were cured in 90 °C for 1 h and well covered with a 24-well plate-sized PDMS. All PDMS substances were washed in ethanol, methanol, and DI water, and UV sterilized for 10 min each before the experiment. A 2 mL of the Nb_2_Se_9_ solution dispersed by poloxamer 188 was filtered on the AAO membrane with a pore diameter of 100 nm to form the solid thin film. The Nb_2_Se_9_ film was dry transferred to the PDMS substrate to evaluate the proliferation of the C2C12 cells on it.

### Live/dead assay and fluorescence imaging

The 2 × 10^4^ cells were seeded in a molded PDMS well plate and incubated for the required time. To avoid cell damage on the substrate, half of the culturing media was replaced every day. The LIVE/DEAD viability/cytotoxicity kit for mammalian cells (Thermo Fisher) was used for fluorescence imaging, in which 20 μL of the supplied ethidium homodimer-1 (EthD-1) was added to 10 mL of PBS and mixed well, followed by the addition of 5 μL of the supplied calcein AM. The cell-culturing media in the samples were removed, and the samples were washed twice with PBS, followed by treatment with the assay solution for 30 min in the dark. An inverted microscope (Olympus, X71) was used for imaging the live and dead cells. Green fluorescence (calcein, excitation (Ex)/emission (Em) =  ~ 494/517 nm) indicated live cells, while red fluorescence (EthD-1, Ex/Em =  ~ 528/617 nm) indicated dead cells^29^.

### Proliferation of C2C12 on substrate

All experiments were conducted using the same method described in the previous section. The MTT assay was conducted with the same method mentioned in the “Proliferation of C2C12 with Nb_2_Se_9_ dispersed by poloxamer 188” section. After the DMSO treatment, formazan solution was aliquoted into the new 96-well plate for the reader^29^.

## Supplementary Information

Supplementary Figures.
