# Author Correction: A study on the bio-applicability of aqueous-dispersed van der Waals 1-D material Nb_2_Se_9_ using poloxamer

**DOI:** 10.1038/s41598-021-88217-4

**Published:** 2021-04-13

**Authors:** Sudong Chae, Seungbae Oh, Kyung Hwan Choi, Jin Woong Lee, Jiho Jeon, Zhixiang Liu, Cong Wang, Changmo Lim, Xue Dong, Chaeheon Woo, Ghulam Asghar, Liyi Shi, Joohoon Kang, Sung Jae Kim, Si Young Song, Jung Heon Lee, Hak Ki Yu, Jae‑Young Choi

**Affiliations:** 1 School of Advanced Materials Science and Engineering, Sungkyunkwan University, Suwon, 16419 Republic of Korea; 2 SKKU Advanced Institute of Nanotechnology (SAINT), Sungkyunkwan University, Suwon, 16419 Republic of Korea; 3 Research Center of Nanoscience and Nanotechnology, Shanghai University, Shanghai, 200444 China; 4 Biomedical Institute for Convergence at SKKU (BICS), Sungkyunkwan University (SKKU), Suwon, Suwon, 16419 Republic of Korea; 5 Department of Orthopaedic Surgery, Dongtan Sacred Heart Hospital, Hwaseong, Republic of Korea; 6 Department of Materials Science and Engineering, Department of Energy Systems Research, Ajou University, Suwon, 16499 Republic of Korea

Correction to: *Scientific Reports* 10.1038/s41598-020-80730-2, published online 08 January 2021

This Article contains a typographical error in the Acknowledgements section.

“This research was supported by Leading University Project for International Cooperation through the National Research Foundation of Korea (NRF) funded by the Ministry of Education (MOE) (NRF-2019R1F1A1063170); and partially supported by NRF-2019R1A2C1006972.”

should read:

“This research was supported by Leading University Project for International Cooperation through the National Research Foundation of Korea (NRF) funded by the Ministry of Education (MOE) (NRF-2019R1A2C2011563); and partially supported by NRF-2019R1A2C1006972.”

