# Supplementary material for: A study on the bio-applicability of aqueous-dispersed van der Waals 1-D material Nb2Se9 using poloxamer
Source: Sci Rep. 2021 Jan 8;11:176. doi: 10.1038/s41598-020-80730-2 (PMC7794490; doi:10.1038/s41598-020-80730-2)
Supplement: Supplementary file 1 — Supplementary Figures. [file 41598_2020_80730_MOESM1_ESM.docx]

**A study on the bio-applicability of aqueous-dispersed van der Waals 1-D material Nb_2_Se_9_ using Poloxamer**

Sudong Chae^1,#^, Seungbae Oh^1,#^, Kyung Hwan Choi^2^, Jin Woong Lee^1^, Jiho Jeon^2^, Zhixiang Liu^2^, Cong Wang^1^, Changmo Lim^1^, Xue Dong^2^, Chaeheon Woo^1^, Ghulam Asghar^2^, Liyi Shi^3^, Joohoon Kang^1^, Sung Jae Kim^5^, Si Young Song^5^, Jung Heon Lee^1,2,4,^*,Hak Ki Yu^6,^*, and Jae-Young Choi^1,2,^*

^1^ School of Advanced Materials Science & Engineering, Sungkyunkwan University, Suwon 16419, Republic of Korea
^2^ SKKU Advanced Institute of Nanotechnology (SAINT), Sungkyunkwan University, Suwon 16419, Republic of Korea

^3^ Research Center of Nanoscience and Nanotechnology, Shanghai University, Shanghai 200444, China

^4^ Biomedical Institute for Convergence at SKKU (BICS), Sungkyunkwan University (SKKU), Suwon, Suwon 16419, Republic of Korea

^5^ Department of Orthopaedic Surgery, Dongtan Sacred Heart Hospital, Hwaseong, Republic of Korea

^6^ Department of Materials Science and Engineering & Department of Energy Systems Research, Ajou University, Suwon, 16499, Republic of Korea

^#^ These authors contributed equally to this work.

*Correspondence and requests for materials should be addressed to J. H. Lee, H. K. Yu and J.-Y. Choi: jhlee7@skku.edu, hakkiyu@ajou.ac.kr & [jy.choi@skku.edu](mailto:jy.choi@skku.edu)


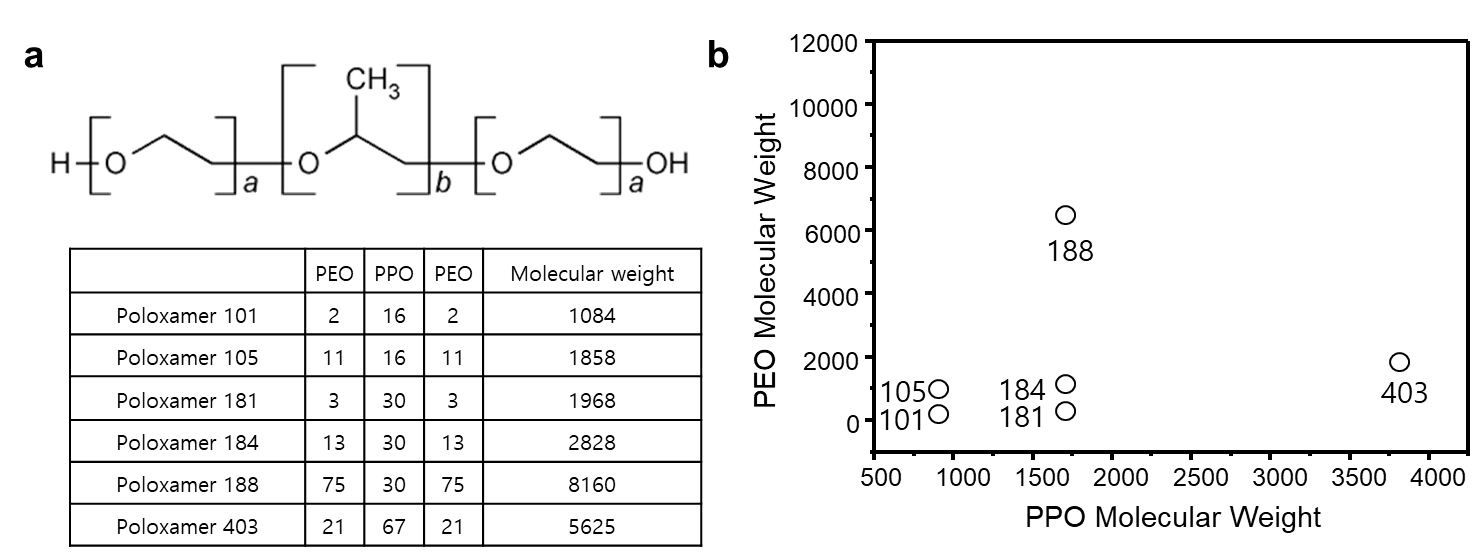


Fig. S1 (a) Molecular structure of Poloxamer and basic information of each poloxamer. (b) PEO and PPO molecular weight distribution of Poloxamers.


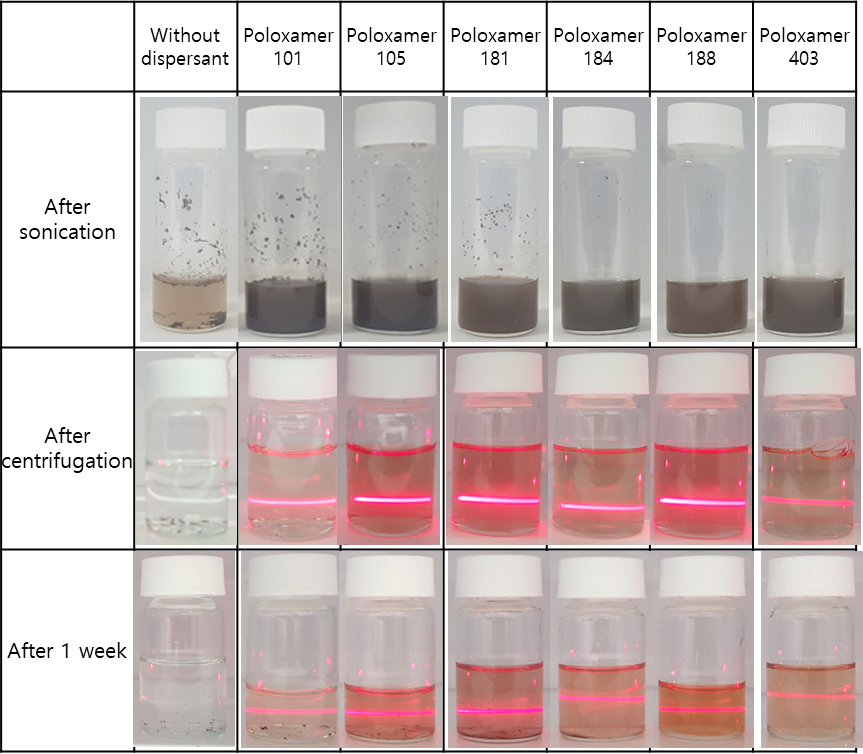


Fig. S2 Photographs after sonication, centrifugation, and a week aging of Nb_2_Se_9_ solution dispersed in water with various Poloxamers.
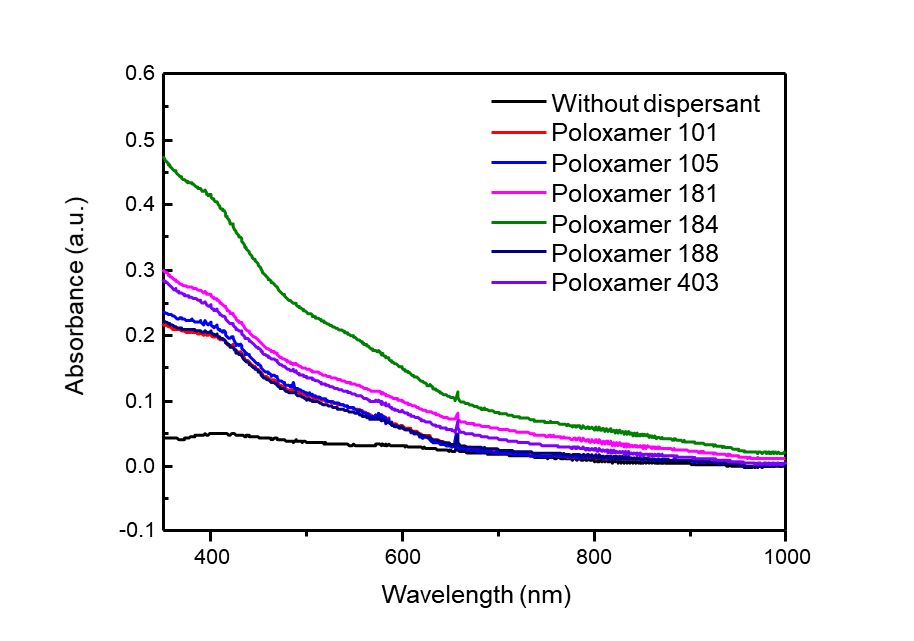


**Fig. S3** UV-vis absorption spectra of Nb_2_Se_9_ dispersed solution in water with various Poloxamers.


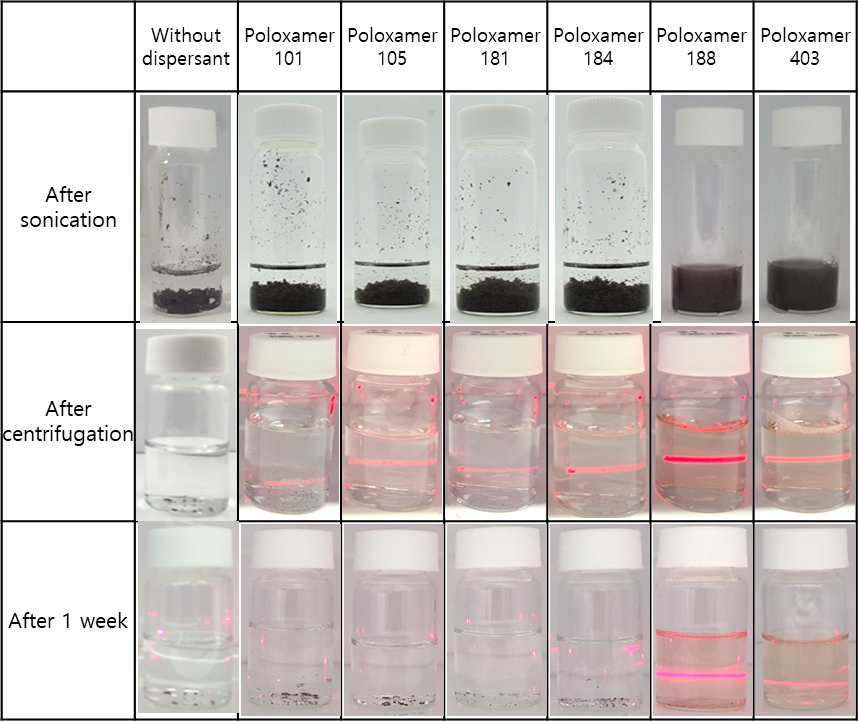


Fig. S4 Photographs after sonication, centrifugation, and a week aging of Nb_2_Se_9_ solution dispersed in PBS buffer solution with various Poloxamers.
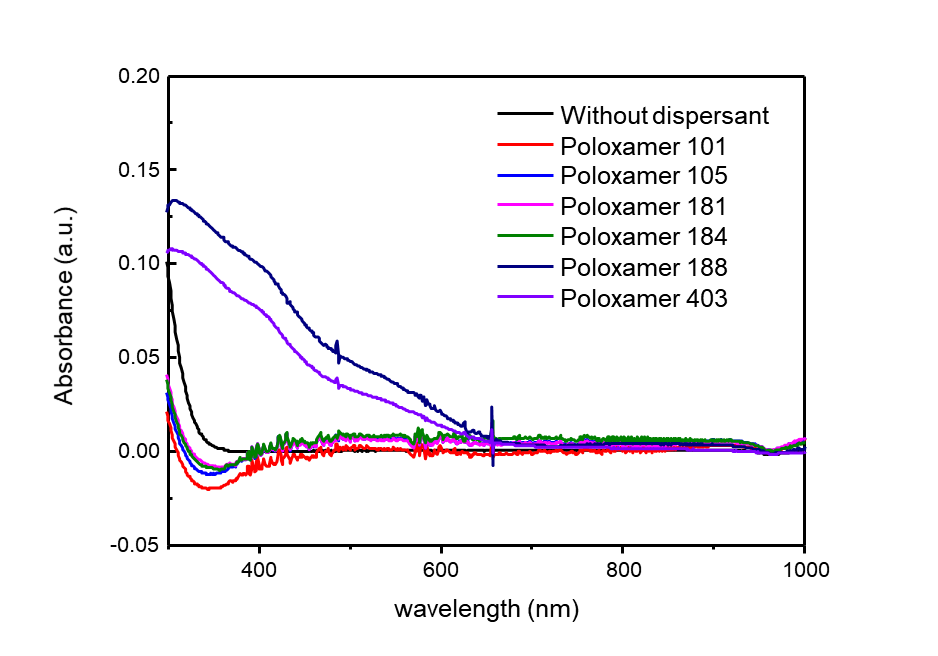
Fig. S5 UV-vis absorption spectra of Nb_2_Se_9_ dispersed solution in PBS buffer solution with various Poloxamers.

**Fig. S6** Zeta potential of Nb2Se9 dispersed solution without and with poloxamer.
